# Clinical effects and pharmacokinetics of nebulized lidocaine in healthy horses

**DOI:** 10.3389/fvets.2022.984108

**Published:** 2022-09-15

**Authors:** Jillian Minuto, Daniela Bedenice, Michelle Ceresia, Iman Zaghloul, Mark Böhlke, Melissa R. Mazan

**Affiliations:** ^1^Department of Clinical Sciences, Cummings School of Veterinary Medicine at Tufts University, North Grafton, MA, United States; ^2^Department of Pharmacy Practice, School of Pharmacy, MCPHS University, Boston, MA, United States; ^3^Department of Pharmaceutical Sciences, School of Pharmacy, MCPHS University, Boston, MA, United States

**Keywords:** nebulize, lidocaine, equine asthma, pharmacokinetics, bronchoalveolar lavage, histamine bronchoprovocation, horse

## Abstract

**Background:**

Nebulized lidocaine appears promising as a novel corticosteroid-sparing therapeutic for equine asthma, but its safety and pharmacokinetic behavior have yet to be confirmed.

**Objective:**

To describe the effect of nebulized lidocaine on upper airway sensitivity, lung mechanics, and lower respiratory cellular response of healthy horses, as well as delivery of lidocaine to lower airways, and its subsequent absorption, clearance, and duration of detectability.

**Animals:**

Six healthy university- and client-owned horses with normal physical examination and serum amyloid A, and no history of respiratory disease within 6 months.

**Methods:**

Prospective, descriptive study evaluating the immediate effects of 1 mg/kg 4% preservative-free lidocaine following nebulization with the Flexineb^®^. Prior to and following nebulization, horses were assessed using upper airway endoscopy, bronchoalveolar lavage, and pulmonary function testing with esophageal balloon/pneumotachography and histamine bronchoprovocation. Additionally, blood and urine were collected at predetermined times following single-dose intravenous and nebulized lidocaine administration for pharmacokinetic analysis.

**Results:**

Upper airway sensitivity was unchanged following lidocaine nebulization, and no laryngospasm or excessive salivation was noted. Lidocaine nebulization (1 mg/kg) resulted in a mean epithelial lining fluid concentration of 9.63 ± 5.05 μg/mL, and a bioavailability of 29.7 ± 7.76%. Lidocaine concentrations were higher in epithelial lining fluid than in systemic circulation (C_max_ 149.23 ± 78.74 μg/L, C_ELF_:C_maxplasma_ 64.4, range 26.5–136.8). Serum and urine lidocaine levels remained detectable for 24 and 48 h, respectively, following nebulization of a single dose. Baseline spirometry, lung resistance and dynamic compliance, remained normal following lidocaine nebulization, with resistance decreasing post-nebulization. Compared to the pre-nebulization group, two additional horses were hyperresponsive following lidocaine nebulization. There was a significant increase in mean airway responsiveness post-lidocaine nebulization, based on lung resistance, but not dynamic compliance. One horse had BAL cytology consistent with airway inflammation both before and after lidocaine treatment.

**Conclusions:**

Nebulized lidocaine was not associated with adverse effects on upper airway sensitivity or BAL cytology. While baseline lung resistance was unchanged, increased airway reactivity to histamine bronchoprovocation in the absence of clinical signs was seen in some horses following nebulization. Further research is necessary to evaluate drug delivery, adverse events, and efficacy in asthmatic horses.

## Introduction

Equine asthma (EA) is endemic in stabled horses of all ages and is a pervasive cause of poor performance. It is characterized by airway inflammation and hyperresponsiveness as well as by increased mucus production, which result in chronic coughing. Corticosteroids are, to date, the best and most common pharmacologic treatment for the airway inflammation that underlies EA; however, systemic corticosteroids have unacceptable long-term adverse effects, while inhaled corticosteroids are expensive and increasingly unavailable. There is an urgent need to explore methods of treatment for horses with EA that decrease inflammation and are safe, inexpensive, readily available, and decrease corticosteroid dependence.

Nebulized lidocaine represents a novel approach to treatment of airway inflammation in EA. Lidocaine is an amide-derivative of diethylaminoacetic acid, a local anesthetic, and a class 1b anti-arrhythmic that is FDA-approved for use in local anesthesia and treatment of ventricular arrhythmias. It is also an Association of Racing Commissioners International (ARCI) Class 2 foreign substance that may cause regulators to impose substantial penalties if residues are identified in post-race samples (1). Lidocaine exerts its anesthetic action by blocking high speed voltage-gated sodium channels (2). It also has immunomodulatory effects by directly regulating inflammation (3) and by silencing nociceptors and decreasing neuro-immune interactions (4). Lidocaine has been used therapeutically and in clinical research for its corticosteroid-sparing effects in human pulmonology for upwards of 50 years, and has shown similar promise in cats for reducing airway resistance and peripheral blood eosinophilia (5). In a recent clinical trial with asthmatic horses in our laboratory, bronchoalveolar lavage (BAL) neutrophilia and tracheal mucus score (TMS) decreased following 14 days of twice-daily lidocaine nebulization (1.0 mg/kg) (6). While lidocaine remains primarily employed as a local anesthetic for bronchoscopy in humans, a recent report demonstrating the effectiveness of lidocaine in ameliorating cough in humans has renewed interest in this modality (7). In addition, more specific and effective lidocaine analogs to treat or prevent life-threatening bronchoconstriction are being developed (8).

Lidocaine suppresses the cough reflex induced by mechanical and chemical stimulation by acting locally to inhibit conduction of afferent nerve impulses and topically anesthetizing the oropharynx and large airways. General numbing of the airways has been reported to provide improved tolerance to respiratory irritants (9). In patients with asthma, inhibition of neural output from receptors in the airways has been shown to prevent interleukin (IL)-5 mediated eosinophil activation by cytokines, minimize IL-3, IL-5 and granulocyte-macrophage colony stimulating factor (GM-CSF) mediated eosinophil survival, and diminish subsequent inflammation of the airways caused by damage to epithelial and smooth muscle cells (10). Furthermore, the effects of lidocaine on IL-3, IL-5, and GM-CSF were reversible, while the effect on interferon (IFN) was only partially overcome, matching the pattern of cytokine inhibition previously reported for glucocorticoids (11). When human peripheral eosinophils were stimulated *in vitro* with IL-3, IL-5 and GM-CSF, superoxide production was decreased in the presence of lidocaine, supporting the role of lidocaine as a potential anti-inflammatory agent (12). Lidocaine has been shown to be effective in improving clinical signs and lung function in mild-to-moderate asthma and attenuating cough in humans (13, 14), improving lung function in asthmatic cats (5), and decreasing airway leukocytes, peribronchial fibrosis, and mucus production in a rodent model of allergic airway disease (4). Nebulized lidocaine alleviated the urge to cough and cough severity in a recent clinical trial in humans with refractory chronic cough, although lidocaine only decreased cough frequency when administered as a topical throat spray (7). In addition, lidocaine is well-suited to nebulization of the airways due to its favorable osmolality and pH, as well as low expense (15, 16).

While nebulized lidocaine at appropriate doses has been shown to be safe and effective in asthmatic humans and cats (5, 9), its safety for use in equine patients has yet to be demonstrated. Lidocaine is commonly used intravenously in equine hospitals to address gastrointestinal pain by decreasing inflammation (17), with a loading dose of 1.3 mg/kg followed by 3.0 mg/kg/h by constant rate infusion with no adverse effects. Meanwhile, human asthma patients (4 mg/kg) and cats (2 mg/kg) have received nebulized lidocaine at higher doses without adverse effect (5, 9, 18). However, the extent to which nebulized lidocaine is deposited in the lung fluid and systemically absorbed in horses is completely unknown. Preliminary work from our laboratory shows support for the anti-inflammatory effect of lidocaine in horses with EA (6); however, important questions regarding the effect of lidocaine on bronchomotor tone, response to bronchoprovocation, cytological evidence of inflammation in bronchoalveolar lavage fluid (BALF), and safety with respect to swallowing function remain unanswered.

Nebulized lidocaine, as an inexpensive, potentially safe, and novel anti-inflammatory and immunomodulatory drug, represents a chance for a real change in treatment of EA. The goal of this study is to establish safety recommendations for use of nebulized lidocaine in asthmatic equine patients. Therefore, our objectives are 2-fold: first, to evaluate lidocaine-associated acute effects on upper and lower airway sensitivity, lung function, and cellular inflammation in BALF of healthy horses and second, to determine the extent of deposition of lidocaine in the lower airways and establish systemic absorption and clearance of nebulized lidocaine.

## Materials and methods

### Experimental design

Ten healthy client- and university-owned adult horses were included in this prospective, descriptive study, with a total of 6 horses completing each of 4 experiments outlined below. Five horses completed intravenous lidocaine pharmacokinetics. Sample size was determined based on convenience sampling, as power analysis was not indicated for descriptive statistics. Horses were considered healthy based on normal physical examination and anamnesis, with no history of lower airway disease within the past 6 months. A prerequisite normal serum amyloid A (SAA, Stable Lab, Zoetis Inc, Kalamazoo MI) was used to minimize the likelihood of occult infectious respiratory disease (3). Three horses with significant behavioral problems that precluded adequate lung function testing were excluded, while a fourth was euthanized due to acute neurologic dysfunction not associated with the current study. Testing took place over a 1-year period, with at least a 1-week washout period between phases. The experimental protocol was approved by the Institutional Animal Care and Use Committee (IACUC) of the Cummings School of Veterinary Medicine at Tufts University and completed following written client consent approved by the Clinical Studies Review Committee (CSRC).

For all nebulization procedures, the portable equine nebulizer Flexineb^®^ (Flexineb North America, Union City TN) was used to produce a fine particulate respirable solution. The particle size of medications given through this mask was previously validated with the appliance and determined to be sufficiently small to reach the lower airways at 4.11 μm mass median diameter (unpublished data, Flexineb). A 4% preservative free lidocaine solution (Hospira, Inc., Lake Forest, IL) was filtered with a 5-micron filter prior to administration of a 1 mg/kg nebulized dose. Condensate that accumulated in the mask during nebulization was collected and quantified.

### Upper airway sensitivity

**Figure 1:**
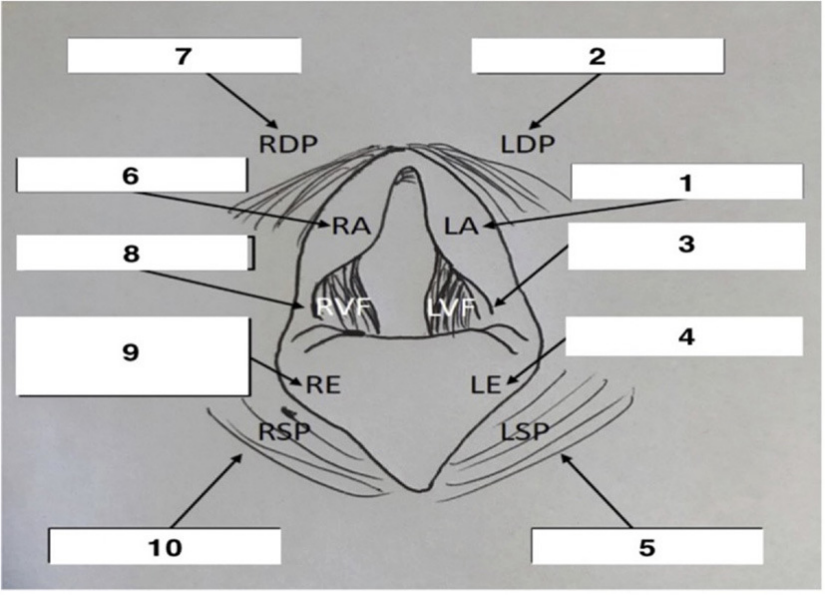
Schematic representation of endoscopic view of the larynx with probe sequence. Adapted from Manneveau (19). Adapted with permission.

Using non-sedated horses, airway endoscopy was performed prior to, and repeated immediately after lidocaine nebulization using a 1-meter video endoscope (1 m length, 12.9 mm outer diameter Endoscope, Fujinon, Wayne, NJ). An atraumatic probe as used to assess upper airway sensitivity and briskness of swallowing. Based on a protocol from Manneveau et al., the larynx was gently probed in the following sequence, first on the left and then the right: arytenoid cartilage, dorsal pharynx, vocal fold, epiglottis, soft palate (19), as outlined in Figure 1. Upper airway abnormalities and number of probes needed to trigger a response were recorded, with a maximum of 5 probes at any one site. If no reaction was observed after five stimulations on the same area, stimulation was stopped and performed on the next area. Tracheal mucus was then graded (0–5), ranging from none to profuse mucus (20), and the number of coughs elicited during tracheal evaluation was recorded. All endoscopies were recorded for blinded review and scoring.

### Lung deposition of nebulized lidocaine

Horses were nebulized with lidocaine, then immediately sedated with xylazine 0.5 mg/kg IV (AnaSed ^Ⓡ^LA 100 mg/mL, MWI, Boise, ID) and butorphanol 0.01 mg/kg IV (Torbugesic^Ⓡ^ 10 mg/mL, Zoetis Inc, Kalamazoo, MI) for BAL using a commercial cuffed BAL tube (Bivona Medical Technologies, Gary, IL). A total of 120 mL of diluted 0.3% mepivacaine (Carbocaine-V 20 mg/ml, Zoetis, Kalamazoo, MI) was used to anesthetize the upper respiratory tract and bronchi during passage of the tube. Once wedged in a bronchus, two aliquots of warmed 250 mL saline were instilled, then aspirated via a suction pump at 10-cm H_2_O (EasyVac, Precision Medical Inc, Northampton, PA). The BALF was placed on ice for no more than 1 h before being centrifuged at 500-g for 10 min at 4°C. Blood samples were placed in red top tubes, allowed to sit on ice for 20 min, then centrifuged at 500-g for 10 min at 4°C. Paired BALF supernatant and serum samples were stored at−80°C until analysis.

Based on the knowledge that urea is freely diffusible through most body compartments, including the lung, and that concentrations are similar in serum and epithelial lining fluid (ELF) and tend to be less affected by pulmonary disorders than other molecules (21, 22), urea was used to estimate the dilution of ELF within BALF. Commercially available ELISA kits (BioChain Institute Inc, Newark, CA) were used to measure serum and BALF urea concentrations, while BALF lidocaine concentrations were determined using high performance liquid chromatography and tandem mass spectrometry. A dilution factor was then calculated to determine the lidocaine concentration in non-diluted ELF using serum obtained immediately prior to BAL (21, 22):


(1) $$\begin{array}{l} ELF\;dilution\;factor\;\left(\%\right)=\;\frac{Urea_{BALF}}{Urea_{Serum}}\times 100 \end{array}$$

The concentration of lidocaine in ELF ([lidocaine]_ELF_) was then derived from the following relationship:


(2) $$\begin{array}{l} {\left[Lidocaine\right]}_{ELF}=\frac{{\left[lidocaine\right]}_{BALF}}{ELF\;dilution\;factor} \end{array}$$

where [lidocaine]_BALF_ is the measured concentration of lidocaine in BALF supernatant.

### Pharmacokinetics

Lidocaine was again administered using a commercially available mask and nebulizer specifically designed for use in horses (Flexineb^Ⓡ^). Blood samples were obtained at 0, 5, 10, 15, 20, 30, 40, 60 min and 2, 4, 6, 12, 18, 24, 48, 72, and 96 h after initiation of nebulization from a catheter placed in a jugular vein prior to nebulization without the aid of local anesthetic. Samples were placed in 3 mL collection tubes containing EDTA. Blood samples were centrifuged at 500 g for 10 min and plasma was stored at−80°C until assayed. Urine samples were collected via free-catch method or by an aseptically placed urinary catheter using standing sedation with xylazine (0.5 mg/kg of body weight IV) administered immediately before sample collection. Concentrations of lidocaine and its major metabolite, monoethylglycinexylidide (MEGX), were determined in urine pre-administration (time = 0) and hourly for the first 24 h, as well as 48, 72, and 96 h after administration of lidocaine. Urine samples for lidocaine and MEGX were collected in 3 mL collecting tubes without anticoagulant and were frozen until analysis at −80°C.

At least 1 week after previous lidocaine administration, 1.3 mg/kg lidocaine was administered intravenously as an infusion over 10 min. Blood samples were again obtained from a catheter placed in a jugular vein at−10 (prior to infusion), 0 (end of infusion), 5, 10, 15, 20, 30, 45, 60 min and 2, 3, 4, 6, 8, 10, and 12 h after drug administration. Collected EDTA whole blood was again centrifuged at 500-g for 10 min and plasma was stored as−80°C until assayed.

#### Drug analysis

Deionized water was purified with a Barnstead™ NANOpure™ water system (Thermo Fisher Scientific, Waltham, MA) which provided 18.2 MΩ-cm water. Methanol and acetonitrile were OmniSolv^®^ from MilliporeSigma (Burlington, MA). Formic acid was Optima^Ⓡ^ LC/MS (Fisher Scientific, Fair Lawn, NJ). Lidocaine was from Sigma Aldrich (Milwaukee, WI) and deuterium-labeled lidocaine (lidocaine-d_10_) was from Cayman Chemical (Ann Arbor, MI). EZ Flow^®^ nylon membranes (0.22 μm pores) used to filter mobile phases were from VWR (Avantor, Radnor, PA). Blank horse plasma and urine for calibration standards were collected from one of the study horses before the study began.

Plasma and BALF samples were analyzed using a high-performance liquid chromatography (HPLC) system consisting of an HP1100 HPLC (Agilent, Santa Clara, CA) coupled to an AB/SCIEX API 4000 triple quadrupole mass spectrometer (AB/SCIEX, Framingham, MA). The column used was a Kinetex^Ⓡ^ PFP (100 x 2.1 mm, 2.6 μm particles, 100 Å pores, Phenomenex, Torrance, CA) and the mobile phase consisted of water/acetonitrile (ACN), 85:15 (v/v) with 0.1% formic acid added to each solvent. The flow rate was 150 μL/min and lidocaine and the internal standard, lidocaine-d_10_ eluted at ~3.4 min.

The mass spectrometer was used with TurboIonSpray™ positive ionization, in multiple reaction monitoring mode. Parameters optimized for lidocaine and lidocaine-d_10_ were curtain gas 20, ion spray voltage (IS) 4000V, probe temperature 550°C, nebulizer gas (GS1) 50, auxiliary gas (GS2) 50, entrance potential 10V and collision energy 27V. The declustering and collision cell exit potentials were 60V and 13.7V for lidocaine and 52V and 15.3V for lidocaine-d_10_. Transitions monitored were 235.3/86.3 for lidocaine and 245.3/96.3 for lidocaine-d_10_. Control of the HPLC system and mass spectrometer and analysis of data were with Analyst 1.6.2 software.

Due to the high concentration of the condensate, samples were analyzed by an HPLC/UV method with a Waters e2695 separations module and a 2489 UV/VIS detector (Waters, Milford, MA). The column used was a Phenomenex Luna C_18_(2) (150 x 4.6 mm, 5 μm particles, 100Å pores) with a mobile phase of 20 mM KH_2_PO_4_ (pH 5.5)/ACN, 65:35 (v/v), with a flow rate of 1.2 mL/min and detection at 230 nm. The retention time of lidocaine with this method was ~7.7 min.

A stock solution of lidocaine was prepared in methanol at 1 mg/mL. A 10 μL aliquot of this solution was added to 90 μL of blank horse plasma or urine which had been analyzed to ensure that no lidocaine was present. From this, dilutions were made in this plasma and urine to appropriate ranges of concentration for calibration. Standards for analysis of BALF samples were prepared in blank BALF.

Aliquots of plasma, urine, and BALF were diluted appropriately with ACN containing lidocaine-d_10_ as an internal standard. These mixtures were centrifuged at 16,162-g for 10 min. Aliquots of the supernatants were diluted with H_2_O/ACN in proportions to produce a final solvent ratio of 1:1 H_2_O/ACN in samples to be injected. These mixtures were centrifuged at 16,162-g for 10 min and 2 μL aliquots of the supernatant were injected into the HPLC system. Standards in the appropriate matrix were prepared at an appropriate range of concentrations to analyze samples collected at different time points and were prepared identically to samples. The lower limit of quantification (LLOQ) was established at 2 ng/mL and the limit of detection (LOD) was 0.5 ng/mL for both lidocaine and MEGX.

#### Pharmacokinetic analyses

Non-compartmental and compartmental pharmacokinetic parameters were calculated using Phoenix™ WinNonlin software v 8.3 (Pharsight Corporation, Mountain View, CA). Plasma concentrations following the inhalation route were used to calculate the non-compartmental parameters: area under the curve (AUC_0−∞_), area under the first moment curve (AUMC_0−∞_), mean residence time (MRT), maximum concentrations (C_max_), time to reach the maximum observed drug concentration (T_max_), apparent volume of distribution (V_z_/F) and apparent clearance (Cl/F), normalized to horse body weight. The linear log trapezoidal method was used in the calculation of AUC. The elimination rate constant was calculated using the “Time Range” method (Slope Selector). The time range is selected in the lambda Z calculation method with the start and end times specified for each subject. To calculate the mean absorption time (MAT), the MRT_neb_ was corrected by subtracting the MRT_iv_. The absorption rate constant was calculated from MAT values for the nebulized lidocaine for horses receiving both IV and nebulized treatments (horse # 1, 4 and 5). The absolute bioavailability of nebulized lidocaine was estimated based on AUC_0−∞_ ratios determined after nebulized lidocaine (1 mg/kg) and intravenous administration (1.3 mg/kg).

The individual plasma concentrations following intravenous administration were best described by a two-compartment model as determined by the coefficients of variation in the estimated parameters, the lowest sum of squares, and Akaike criteria. The distribution (α and T_1/2α_) and elimination (β and T_1/2β_) phases were calculated using the two-compartment first-order equation


(3) $$\begin{array}{l} C_{t}=Ae^{-\alpha t}+Be^{-\beta t} \end{array}$$

where the values of A and B are the extrapolated concentrations to time 0 of the distribution and elimination phases. The area under the plasma concentration–time curve (AUC_0−∞_) and the area under the first moment curve (AUMC_0−∞_) were determined using the trapezoidal method with extrapolation to infinity. The systemic clearance (CL) was calculated using the dose/AUC, and the mean residence time (MRT) was obtained from the ratio of AUMC_0−∞_ to AUC_0−∞_. The volume of distribution of the central compartment (V_c_) was calculated from the dose/C_0_ (where C_0_ = A + B). The volume of distribution area (V_dβ_) was also determined from CL/β. The apparent steady-state volume of distribution (V_dss_ = CL × MRT) was calculated. Pharmacokinetic parameters are expressed as mean ± standard deviation ($\bar{X}\pm$SD).

### Lung function testing

Horses were assessed on two consecutive days, the first without prior lidocaine nebulization and the second within minutes of completion of lidocaine nebulization. Horses were sedated using 0.1–0.5 mg/kg IV xylazine prior to naso-esophageal intubation for balloon placement in the mid-thoracic region, then allowed 30 min to recover from sedation prior to assessment. On day two, nebulization was initiated following this recovery period. Lung function was then evaluated as previously described (23). In brief, the esophageal balloon catheter was connected to a pressure transducer for estimation of transpulmonary pressure and a pneumotachograph (Fleisch #5, EMMS, Hampshire, UK) attached to an airtight mask was connected to a differential pressure transducer (Emka USB B_T differential sensor along with amplifiers EMKA/SciReq, Model USB_AMP_4BR) for determination of flow and volume by the electronic integration of flow with respect to time. Pulmonary mechanics analysis software (Emka Iox 2 Software Suite, Emka Technologies, Falls Church, VA) was then used to record pressure, flow, and volume on a breath-by-breath basis. The balloon was checked for leaks periodically throughout the study. Pulmonary resistance (R_L_) and dynamic compliance (C_dyn_) were calculated by the lung single compartment model, using the isovolumetric method with dedicated software (Emka Iox 2 Software Suite, Emka Technologies, Falls Church, VA).

#### Histamine bronchoprovocation

To detect changes in airway responsiveness associated with lidocaine nebulization, histamine bronchoprovocation (HBP) was employed. Nebulization of 2 mL of 0.9% saline (normal saline solution, VEDCO Inc, St. Joseph, MO) over 2 min using the same low dead-space face mask with a portable air compressor and nebulizer (NE-C801 CompAIR Compressor Nebulizer System, Omron Healthcare Co, Lake Forest, IL) was performed and repeated for subsequent increasing concentrations of histamine (histamine bisphosphate monohydrate, Sigma-Aldrich, St. Louis, MO) in saline solution (2, 4, 8, and 16 mg/mL). Lung function measurements were repeated following each nebulized dose until either a 100% increase in R_L_ from saline baseline (PC_100_R_L_) was achieved, or the horse displayed a clinical reaction to histamine administration, such as a notably increased respiratory rate or effort, or repeated coughing. Following HBP, a dose-response curve was generated to determine the histamine dose required to reach a 75% increase in saline baseline resistance (PC_75_R_L_) and 35% decrease in dynamic compliance (PC_35_C_dyn_) for each horse, whereby a lower provocative concentration indicates greater airway responsiveness. Clinically, horses with PC_35_C_dyn_ and PC_75_R_L_ <6 mg/mL of histamine are considered hyperresponsive and those with PC_35_C_dyn_ and PC_75_R_L_ >8 mg/mL are considered non-responsive, while those with provocative concentrations of 6–8 mg/mL of histamine fall into a diagnostic gray zone. For statistical analysis, horses with PC_35_C_dyn_ and PC_75_R_L_ <6 mg/mL were considered responsive, while horses with PC_35_C_dyn_ and PC_75_R_L_ >6 mg/mL were considered non-responsive. This procedure was repeated on the 2nd day following lidocaine nebulization.

### Airway cytology

At the conclusion of HBP each day horses were nebulized with 0.005 mg/kg albuterol (albuterol sulfate 0.083%, Nephron Pharmaceuticals Corporation, West Columbia, SC) and allowed a 15-min waiting period to reduce any residual bronchoconstrictive effects of histamine prior to BAL. Horses were then sedated (xylazine 0.5 mg/kg IV, butorphanol 0.01 mg/kg IV) for BAL, as previously described, to assess the effect of nebulized lidocaine on the cellular inflammatory response. BALF was kept on ice until it was delivered to the laboratory within 30 min, where it was centrifuged at 500 g for 10 min at 4°C. Slides were then prepared using the sedimentation method and stained with modified Wright-Giemsa (Dip Quik, Jorgensen Laboratories, Loveland, CO) and toluidine blue (Electron Microscopy Sciences, Hatfield, PA) stains, the latter for the enumeration of mast cells. Cells (*n* = 500 Wright-Giemsa, *n* = 1,000 toluidine blue) were classified by two investigators (JM, MM) as the percentage of total cells that were macrophages, lymphocytes, neutrophils, eosinophils, and mast cells (400x magnification). Horses were determined to have an abnormal BALF cytology using cutoff values established by our laboratory (> 10% neutrophils, > 2% mast cells, or ≥ 1% eosinophils) (24).

### Data analysis

Data were compiled in Microsoft Excel^Ⓡ^, with statistical assessment performed in SPSS (IBM Corp, SPSS Statistics for Windows, Version 28.0, Armonk, NY). Normality was assessed based on visual assessment of histograms of the differences in data pre- and post-lidocaine administration. Hypothesis-based testing of normality, such as the Shapiro-Wilk test, was considered unreliable, given the small sample size. For this same reason, non-parametric testing was considered most appropriate. Differences in airway sensitivity, lung function, and BAL cytology before and after lidocaine nebulization were assessed using the Mann-Whitney U test, with *p* < 0.05 denoting significance. However, in order for the effect to be statistically significant, this study would have required 258 horses, based on a power calculation of 80% for all measures. All paired data were reported as the median ± interquartile range, while pharmacokinetic data were reported as mean ± standard deviation for convention.

## Results

### Horses

Average weight of the horses was 495 ± 59 kg, and median age was 19 ± 13 yrs. The study population consisted of Quarter Horses (5/10), Standardbreds (2/10), Paint Horses (2/10), and one Morgan (1/10), including 5 mares and 5 geldings. Two of 10 horses were in a low intensity exercise program. Horses inhaled 12.32 ± 2.27 mL nebulized lidocaine over a period of 9.33 to 28.50 min (17.05 ± 5.45 min), creating a median volume of 3.95 mL condensate (IQR = 3.50 mL) within the nebulization chamber. The exhaled condensate had a mean lidocaine concentration of 37.99 ± 8.38 mg/mL, comparable to the concentration of the original nebulized solution (40 mg/mL). No immediate side-effects, such as hypersalivation, were appreciated during nebulization. However, one horse coughed post-nebulization in 2 out of 4 experiments. Four of the 10 horses had either excessive TMS, airway hyperreactivity (AHR), or abnormal BAL cytology on baseline evaluation, although they remained within the defined inclusion criteria. One horse had both excessive TMS and AHR. A summary of baseline diagnostics is outlined in Table 1.

**Table 1:** Outcomes of EA diagnostic criteria, including tracheal mucus score, BAL cytology, and histamine bronchoprovocation, before and after lidocaine nebulization.

	**Tracheal mucus score**		**BAL cytology (%)**						**Histamine bronchoprovocation**			
			**PMN**		**Eosinophils**		**Mast Cells**		**PC**_**35**_ **C**_**dyn**_		**PC**_**75**_ **R**_**L**_	
**Horse**	**Pre**	**Post**	**Pre**	**Post**	**Pre**	**Post**	**Pre**	**Post**	**Pre**	**Post**	**Pre**	**Post**
1	1	-	0.00	5.00	0.00	0.40	1.00	1.50	20.62	8.46	32.82	30.18
2	**3**	**2**	0.00	3.00	0.00	3.00	0.90	1.40	**4.44**	**5.01**	22.98	6.67
3	-	-	8.39	4.90	0.00	0.00	1.00	1.09	**3.62**	**4.68**	11.26	**3.53**
4	0	0	-	-	-	-	-	-	-	-	-	-
5	1	**2**	9.00	4.00	0.00	0.00	0.50	1.60	35.50	**2.06**	22.10	6.65
6	0	**2**	0.39	2.80	0.00	0.00	1.15	0.83	**1.89**	**1.41**	**2.27**	**1.74**
7	0	0	-	-	-	-	-	-	-	-	-	-
8	-	-	8.18	8.00	**1.80**	**1.60**	1.08	1.09	7.03	8.16	48.47	8.28

Bold denotes clinically abnormal values (TMS ≥ 2, PMN > 10%, Eosinophil > 1%, Mast Cell > 2%, PC_35_C_dyn_ < 6.0, PC_75_R_L_ <6.0) - denotes diagnostics not assessed p > 0.05 for all groups.

### Upper airway sensitivity

Prior to nebulization, 0/6 horses had pharyngeal collapse, while 4/6 had transient dorsal displacement of the soft palate. Two of six horses had mild arytenoid asynchrony (grade 2 of 4), while the remaining horses were normal. Following nebulization, there was no change in the occurrence of pharyngeal collapse (0/6), and the incidence of displacement of the soft palate decreased (1/6). Only one of the 2 previously observed horses continued to show mild arytenoid asynchrony (grade 2 of 4), while the remaining horses were normal (5/6). No structural upper airway abnormalities were noted prior to or after nebulization. Cumulative probes per horse pre- and post-nebulization were not different [pre: 21 ± 10 (14–3), post: 22 ± 13 (13–43), *p* = 0.872]. There was no difference in the cumulative number of probes over time across the two groups (Pre: 139, Post: 143). Responses to stimulation of individual sites are summarized in Table 2. Pre-nebulization, 4/6 horses coughed during tracheoscopy (2 ± 3 coughs, range 0–8), while 3/6 horses coughed post-nebulization (1 ± 4 coughs, range 0–4, *p* = 0.818). Tracheal mucus scores are summarized in Table 1.

**Table 2:** Number of probes required to stimulate swallow response before and after nebulization with 1 mg/kg 4% Lidocaine.

	**Left, pre-nebulization**	**Left, post-nebulization**	**Right, pre-nebulization**	**Right, post-nebulization**
Arytenoid cartilage	1 ± 0	1 ± 0	1 ± 1	2 ± 4
Dorsal pharynx	2 ± 1	1 ± 0	2 ± 2	4 ± 3
Vocal fold	1 ± 2	2 ± 2	1 ± 4	2 ± 1
Epiglottis	2 ± 2	1 ± 1	2 ± 0	2 ± 2
Soft palate	4 ± 3	2 ± 2	3 ± 3	4 ± 3

Maximum number of probes per site 5, with 6 representing no response to maximum number of stimulations. Shown as median ± IQR.

### Lung deposition of nebulized lidocaine

BAL with 500 mL saline yielded returns of 175–370 mL BALF. Based on serum and BALF urea concentrations, ELF comprised 1.00–2.38% of the total yield, equivalent to 2.07–7.13 mL ELF sampled per total BAL volume retrieved. Lidocaine reached a mean concentration of 142.83 ± 34.25 ng/mL BALF, corresponding to a concentration of 9.63 ± 5.05 μg/mL in ELF.

### Pharmacokinetics

**Figure 2:**
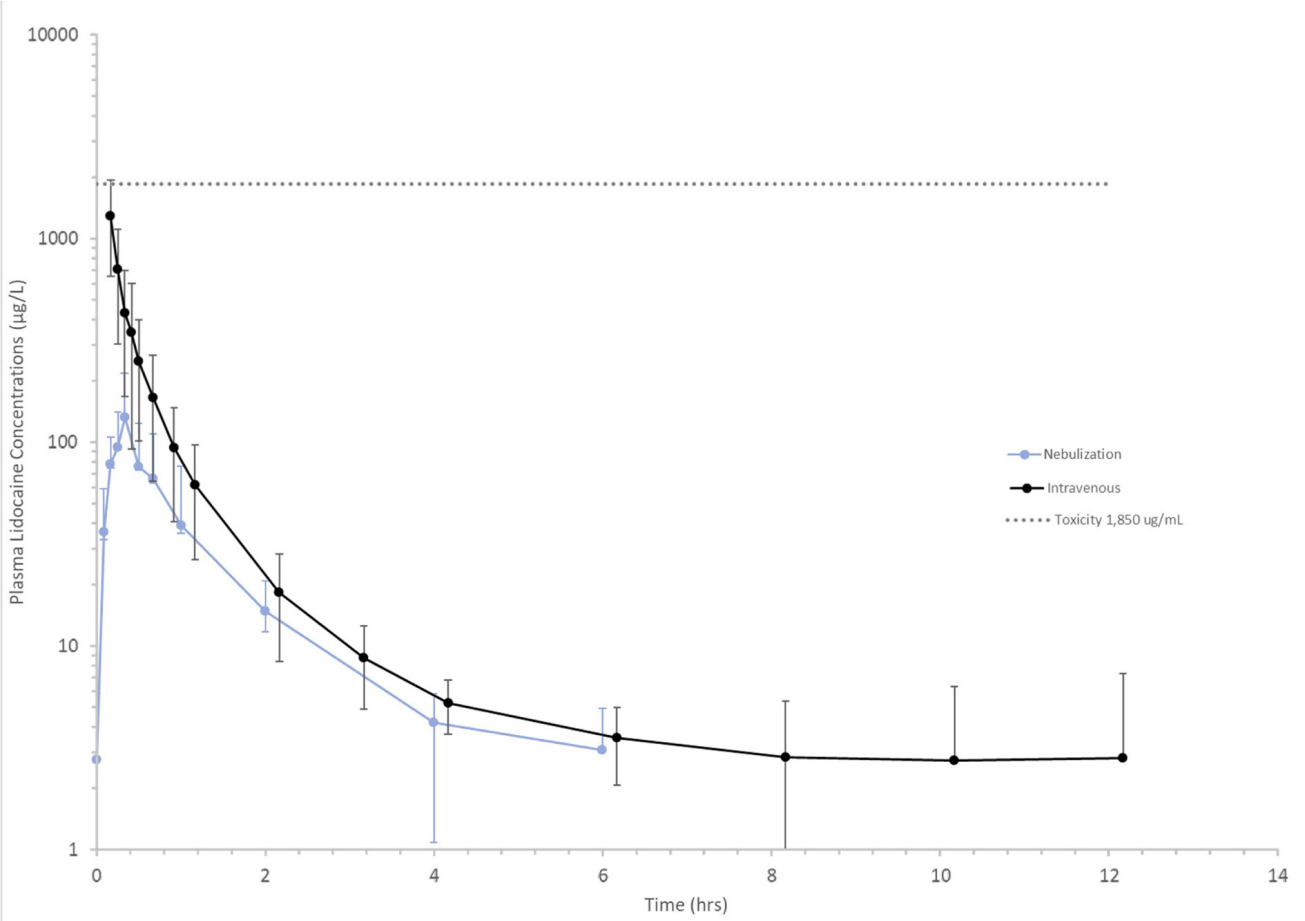
Mean lidocaine plasma concentrations vs. time following a 1.3 mg/kg intravenous (black line) and 1 mg/kg nebulized (blue line) dose in healthy horses. Toxicity (dotted gray) line represents lower end of the lidocaine concentration range at which clinical signs of intoxication have been observed 1,850 μg/L (25).

Following nebulization for the pharmacokinetic study, the mean condensate volume collected was 4.37 ± 2.33 mL (range 1.7–6.8 mL). The mean condensate lidocaine concentration was 38.38 ± 2.42 mg/mL for these horses. Accounting for drug loss in condensate, the mean adjusted administered dose was 302.92 ± 93.30 mg (0.64 ± 0.19 mg/kg). The plasma concentration-time curves and pharmacokinetic parameters following administration of 1.0 mg/kg nebulized and 1.3 mg/kg IV lidocaine in healthy adult horses are represented in Figure 2 and Tables 3, 4. Following nebulization, lidocaine remained quantifiable (LLOQ 2 ng/mL) in blood for 4 h (2/6 horses), 6 h (3/6 horses) and 12 h (1/6 horses) and in urine up to 24 h for most horses. The metabolite, MEGX was quantifiable for 24 h in urine for all of the horses except one (48 h) and detectable (LOD 0.5 ng/mL) for up to 96 h in the urine of 2/6 treated horses.

**Table 3:** Pharmacokinetic parameters of lidocaine following a single intravenous 1.3 mg/kg dose in 5 healthy adult horses.

**Pharmacokinetic parameters**	**Intravenous**
C_0_ (μg/L)	1,388.87 ± 451.74
A (μg/L)	1,371.03 ± 450.28
α (h^−1^)	2.86 ± 0.63
B (μg/L)	17.84 ± 5.83
β (h_−1_)	0.26 ± 0.04
t_1/2α_ (h)	0.25 ± 0.05
t_1/2β_ (h)	2.71 ± 0.47
AUC_0−∞_ (μg*h/L)	549.41 ± 141.99
AUMC_0−∞_ (μg*h^2^/L)	433.74 ± 78.09
CL (L/h/kg)	2.49 ± 0.59
MRT (h)	0.81 ± 0.13
V_c_ (L/kg)	1.01 ± 0.31
V_dβ_ (L/kg)	1.03 ± 0.49
V_dss_ (L/kg)	2.05 ± 0.65
K_10_ (h^−1^)	2.54 ± 0.56
K_12_ (h^−1^)	0.29 ± 0.09
K_21_ (h^−1^)	0.29 ± 0.04

A, extrapolated concentrations to time 0 of the distribution phase; α, rate constant of distribution phase; B, extrapolated concentrations to time 0 of the elimination phase; β, rate constant of elimination phase; t_½α_, distribution half-life; t_½β_ elimination half-life; AUC _0−∞_, area under the plasma concentration time curve from 0 to infinity; AUMC _0−∞_, area under the first moment curve from 0 to infinity; CL, systemic clearance; MRT, mean residence time; V_dss_, volume of distribution at steady state, V_c_, volume of distribution in the central compartment; V_dβ_, volume of distribution during the elimination phase; C_0_, initial concentration; K_10_, microconstant representing drug absorption into the central compartment; K_12_, and K_21_, microconstants representing distribution between the central and peripheral (2^nd^) compartment. Data reported as mean ± standard deviation.

**Table 4:** Pharmacokinetic parameters of lidocaine following a single nebulized 1.0 mg/kg dose in 6 healthy adult horses.

**Pharmacokinetic parameters**	**Nebulization**
C_max_ (μg/L)	149.23 ± 78.74
T_max_ (h)	0.36 ± 0.16
λ (h^−1^)	0.51 ± 0.23
t_½λ_ (h)	1.74 ± 1.10
AUC_0−last_ (μg*h/L)	118.20 ± 55.66
AUC_0−∞_ (μg*h/L)	125.14 ± 55.56
AUC (%extrapolated)	6.6 ± 4.96
AUMC_0−last_ (μg*h^2^/L)	176.14 ± 122.98
AUMC_0−∞_ (μg*h^2^/L)	250.79 ± 192.49
MRT_0−last_ (h)	1.48 ± 0.62
MRT_0−∞_ (h)	2.0 ± 1.05
F (%)	29.70 ± 7.76^†^ 46.4^*^
C_ELF_:C_maxplasma_	64.4

C_max_, maximum concentration; T_max_, time to maximum concentration; λ, elimination rate constant; t_½λ_, elimination half-life; AUC_0−last_, area under the plasma concentration time curve from 0 to the last measurable concentration; AUC _0−∞_, area under the plasma concentration time curve from 0 to infinity; AUC (% extrapolated), percent area estimated from the last measurable concentration to infinity; AUMC _0−last_, area under the first moment curve from 0 to the last measurable concentration; AUMC _0−∞_, area under the first moment curve from 0 to infinity; CL, systemic clearance; MRT_0−last_, mean residence time from 0 to last the measurable concentration; MRT_0−∞_, mean residence time from 0 to infinity; F, bioavailability; C_ELF_:C_Maxplasma_, ratio of the estimated concentration in the epithelial lining fluid compared to the maximum concentration in the plasma.

† Average of horses 1, 4 and 5;

* Calculated using the mean AUC_neb_ and AUC_IV_ of all horses Data reported as mean ± standard deviation.

### Lidocaine detection assay

Intra-day variation for samples with concentrations in the calibration range of 8–2,000 ng/mL ranged from 0.42 to 1.94% while inter-day variation was 0.37–2.68%. For samples with concentrations in the range of 2–100 ng/mL, intra-day variation was 0.62–1.62%, while inter-day variation was 0.77–12.9%.

### Lung function testing

Baseline respiratory rate, tidal volume, minute ventilation, R_L_ and C_dyn_ were within normal ranges, and remained so after lidocaine nebulization, with no significant differences between the two time points. Spirometry and pulmonary function findings are summarized in Table 5.

**Table 5:** Spirometry and esophageal balloon data in 6 healthy horses prior to and immediately following nebulization with 1 mg/kg lidocaine.

	**Pre-lidocaine**	**Post-lidocaine**	***p*-value**
Respiratory rate (br/min)	11 ± 3	10 ± 2	0.49
Tidal volume (L)	7.9 ± 2.6	7.3 ± 2.0	0.93
Minute ventilation (L/min)	79.3 ± 30.4	77.1 ± 19.8	0.82
R_L_ (cmH_2_O/L/sec)	0.31 ± 0.07	0.23 ± 0.05	0.07
C_dyn_ (mL/cmH_2_0)	1.95 ± 0.94	1.83 ± 0.57	0.70
dPpl	4.2 ± 1.3	4.0 ± 0.8	0.70

Shown as median ± IQR. P-value of Mann Whitney U test, p < 0.05 denotes significance. R_L_, lung resistance; Cdyn, dynamic compliance; dPpl, change in pleural pressure.

Five of 6 horses completed HBP testing both before and after nebulization with lidocaine, including the maximum histamine dose of 16 mg/mL. The same subject reached PC_100_R_L_ and showed increased respiratory effort following 4 mg/mL histamine nebulization at both timepoints. Pre-lidocaine, tidal volume increased throughout HBP in the five horses that completed testing (ΔTV = 1.25 ± 0.27 L), while the horse that exceeded PC_100_R_L_ after 4 mg/mL histamine showed a precipitous decline in TV (9.71 to 1.80 L). Following lidocaine, 2/4 horses that had AHR based on PC_35_C_dyn_ showed increases in respiratory rate exceeding 20 breaths per minute (21–25 br/min) and declines in tidal volume (ΔTV = −3.49 to−3.54 L) prior to cessation of bronchoprovocation. Outcomes of HBP are summarized in Tables 1, 6.

**Table 6:** PC_35_C_dyn_ and PC_75_R_L_ before and after nebulized lidocaine administration in 6 healthy horses with and without baseline airway hyperresponsiveness (AHR).

	**PC**_**35**_ **C**_**dyn**_				**PC**_**75**_ **R**_**L**_			
	**Pre**	**Post**	**% change**	***n* =**	**Pre**	**Post**	**% change**	***n* =**
Pre-lidocaine AHR	3.62 ± 1.28	3.37 ± 2.87	12.74 ± 27.19	3	2.27 ± 0	2.63 ± 0.90	−23.56%± 0	1
Pre-lidocaine non-reactors	20.62 ± 14.24	8.31 ± 0.15	−59.0 ± 55.13	3	22.98 ± 10.72	7.47 ± 7.09	−69.9 ± 2.3	5

PC_35_C_dyn_ and PC_75_R_L_ are expressed in mg/mL histamine. AHR defined as PC_35_C_dyn_ or PC_75_R_L_ < 6 mg/mL histamine. Non-reactors defined as PC_35_C_dyn_ or PC_75_R_L_ > 6 mg/mL histamine. Shown as median ± IQR.

### Airway cytology

BAL cytological findings are summarized in Table 1. Out of 6 horses, one had cytological evidence of lower airway inflammation consistent with EA (>1% eosinophils) in BAL performed both before and immediately after nebulization with preservative free lidocaine. No qualitative changes in cell morphology were present following lidocaine nebulization.

## Discussion

In this study we used upper airway endoscopy, BAL, and pulmonary function testing to describe the effect of nebulized lidocaine on the upper airway sensitivity, lung mechanics, and the lower respiratory cellular response of clinically healthy horses, as well as the delivery of lidocaine to the lower airways. Pharmacokinetic assessment was used to outline nebulized lidocaine's subsequent absorption, clearance, and duration of detectability in healthy equine patients. There were seven key findings, discussed below. First, and most importantly, single dose nebulized lidocaine yielded no immediate adverse clinical effects or changes in upper airway sensitivity. In addition, lidocaine concentration in ELF exceeded the anti-arrhythmic therapeutic concentration of 1.5–5.0 μg/mL (26), and thus was present locally at levels that could have pharmacologic effects. Nebulized lidocaine had high bioavailability relative to other nebulized medications, and remained detectable in blood and urine for up to 24- and 48-h post-nebulization, while MEGX remained detectable in urine at 96 h in 2 out of 6 horses. While baseline lung resistance decreased following lidocaine nebulization, an increased number of horses developed AHR in response to HBP, with a significant decrease in provocative histamine concentrations needed to increase lung resistance by 75%. This increased airway responsiveness was most notable in the horses that were non-reactive prior to nebulization. No immediate idiosyncratic effect on BAL cytology was present.

No immediate adverse clinical effects or changes in upper airway sensitivity were present following single dose nebulized lidocaine. There was no difference in the cumulative number of stimulations across 10 sites within the pharynx and larynx needed to induce swallowing prior to or following lidocaine nebulization. Nebulized lidocaine has been used in human medicine to anesthetize the nasal mucosa, pharynx, and larynx prior to bronchoscopy, as well as ancillary therapy to minimize postoperative laryngospasm in children and adults (27, 28). In addition, topical lidocaine minimizes cough during bronchoscopy and BAL in horses (29). Such uses have raised concern for the duration and severity of laryngeal anesthesia achieved in the horse, and its implication for increased risk of aspiration of feed material and possible decreased clearance from the proximal airways. Concerns for temporary desensitization of the pharynx and larynx with risk of dysphagia have led to guidelines in human medicine to avoid eating for 45–60 min after nebulization (14). In a study of long-term safety of nebulized lidocaine in humans, nine percent of reported adverse effects included choking on food or water after nebulization (9). Chinn et al. 1977 reported loss of gag reflex for 45–60 min after intermittent positive pressure breathing, but not following ultrasonic nebulization (18). In another study, cough and gag reflexes were absent in all patients for 15–20 min following delivery of 4% aerosolized lidocaine (30). Despite being counseled to keep horses from eating or drinking for 45 min post-lidocaine nebulization in our recent study (6), owners were remarkably non-compliant; nonetheless, no adverse events were reported. In the present study, right-sided locations (arytenoid cartilages, dorsal pharynx) appeared to require an increased number of stimulations after nebulization, but this difference failed to reach statistical significance. As flow rate and volume of flow should be symmetrical throughout the proximal airways, the increased time between nebulization and stimulation of these right-sided sites compared to the left is considered the most likely justification for a unilateral decrease in sensitivity. Lidocaine takes <1 min to exert local anesthetic effects on the larynx when sprayed topically (31, 32). Even though lidocaine has such a rapid onset of action, the lower local concentrations achieved via nebulization vs. a topical spray makes it likely that an extended contact time is required for the drug to exert its full effect. This question could have been more completely answered by randomizing the order in which upper airway sites were stimulated prior to and after nebulization.

In addition, TMS increased in 2/6 horses following nebulization. This is possibly due to mobilization of mucus from the distal airways following coughing during pre-nebulization tracheoscopy. An immediate release of mucin from goblet cells is considered less likely, as TMS in healthy horses have been shown to remain unchanged after 6–48 h of exposure to dust and allergens during environmental challenge (33).

To best understand the extent of drug delivery and absorption for dosing recommendations, lidocaine concentrations in ELF and plasma were assessed. Plasma and urine concentrations of lidocaine and its active metabolite MEGX were then followed to understand not only its pharmacokinetics when administered by this novel route, but the length of time that lidocaine remained detectable for competition purposes. Using urea as an endogenous marker of BALF dilution, ELF comprised 1.00–2.38% of the total yield, comparable to human studies, where 1 ± 0.1 mL ELF is recovered per 100 mLBALF (21). Urea is a small (60 Daltons) molecule that diffuses freely through the body, including the alveolar wall. As such, it can be used as an endogenous marker to estimate dilution and quantify ELF recovery. However, inconsistent saline dwell times in the distal airways can allow additional urea diffusion, resulting in an overestimation of ELF recovery, and underestimation of drug delivery (21, 25). Possible explanations of such over-estimation include contamination of the BAL tube through the nares, as well as tracheal and upper airway secretions during passage to the tracheobronchial tree, pharynx, trachea, and large bronchi. Additionally, as most blind BAL tubes end up in terminal segmental bronchi in the caudo-dorsal lung, and inhaled particles most often distribute ventrally, it is further possible that the lidocaine concentrations found here are under-reported, although they remain consistent with other nebulized medications reported in the horse (34). Assuming accurate correction for dilution, lidocaine reached concentrations exceeding 9 μg/mL in the epithelial lining fluid of nebulized healthy horses, comparable to or exceeding that of previous publications (35). The favorable ELF to plasma concentration ratio (C_ELF_:C_maxplasma_) indicates that the drug had a comparatively higher concentration at the site of action compared to maximum systemic levels. In addition to exceeding the systemic anti-arrhythmic therapeutic concentration and plasma concentrations obtained during standard continuous rate infusion in horses, ELF lidocaine concentration also exceeded the concentration required in human BALF to inhibit eosinophil survival (10, 36). Meanwhile, peak serum concentrations remained < 0.25 μg/mL, well below concentrations where toxic effects have been reported in humans (8–15 μg/mL), and horses (1.9–4.5 μg/mL) (37, 38). Peak serum concentrations following lidocaine nebulization in humans have been inconsequential, reaching only 1.4 μg/mL with nebulized doses 5-fold above those evaluated here (28). Lidocaine remained detectable in blood and urine for 24- and 48-h post-nebulization, and MEGX remained detectable in urine at 96 h. As xylazine sedative was required for safe urinary catheterization, it must be considered that its action as an ADH antagonist decreased urine concentration and thus could have decreased the concentrations of lidocaine and MEGX obtained, and thus their duration of detectability. While this may have contributed to a dampened maximal urine lidocaine concentration in the 1–3 h post-nebulization, this is considered unlikely to have changed the duration that lidocaine and MEGX were detectable in urine, as xylazine requires 30–60 min to cause a significant increase in the rate of urine production (39), and urinary catheterization was completed within 10 min of xylazine administration. As drug deposition in the distal airways is impeded by modifications in breathing pattern, cough, bronchoconstriction, and airway secretions such as mucus often present in EA (40), investigation into drug delivery in clinically affected animals remains essential to ensure that therapeutic concentrations are achieved. However, the peak serum concentrations achieved in healthy horses here indicate that nebulized lidocaine dosages in excess of 1 mg/kg may be safely considered, if needed to achieve clinical efficacy.

Nebulized lidocaine was rapidly absorbed, achieving C_max_ at 0.36 h. Labedzki *et al* also found in humans that ultrasonic nebulized lidocaine reached C_max_ at 0.68 h. The bioavailability of nebulized lidocaine in this study is considerably higher than in humans (8%) when lidocaine was administered by ultrasonic nebulization (28, 41). The terminal half-life of lidocaine (2.71 ± 0.47 h) following intravenous lidocaine administration was similar to previous reports in horses (42). Likewise, the AUC normalized by dose calculated here agreed with both Engelking et al. (330.2) and Soma et al. (334.7). Additionally, the systemic clearance found here is comparable to previous studies (3.12 ± 0.702 and 2.30 L/h/kg for Engelking et al. and Soma et al., respectively). As the absolute volume of epithelial lining fluid in the horse has not been reported, nuclear scintigraphy would ultimately be required to report the percentage of nebulized drug delivered to the distal airways. When extrapolating from human and ovine data (43, 44), if epithelial lining fluid volume is presumed to be 0.45 mL/kg, the percentage of nebulized drug to reach the distal airways is small at 0.43 +/-0.23%. Thus, at T_max_ the average 500 kg horse receiving 1 mg/kg nebulized lidocaine would then have 2.16 mg of lidocaine within the ELF while 3.73 mg circulated within their 25 L plasma volume. When considering both the increased bioavailability and the low levels of nebulized lidocaine that reach the distal airways, additional sources of lidocaine absorption must be considered. In general, only 10% or less of nebulized medications reach the distal airways (45), with the majority being deposited in the proximal airways where it can be swallowed, cleared by the mucocilliary apparatus, or absorbed transmucosally. Generally, lidocaine is not administered orally due to its significant first pass effect and low bioavailability *via* this route (35%) (26). Meanwhile, the surface area of the equine nasopharynx is immense, exceeding that of humans even in 2- to 3-month-old foals (46, 47). Thus, it is possible that while nebulized lidocaine reaches therapeutic concentrations in the distal airways, most of the systemic absorption comes from transmucosal absorption in the nasopharynx. In support of this, previous studies of inhaled drugs in horses demonstrate that much of the delivered drug is deposited on the extensive nasal mucosa (48).

Assessment of lung function prior to and following lidocaine nebulization was employed to screen for bronchoconstriction, as short-term dose-dependent bronchoconstriction has been noted after lidocaine nebulization in asthmatic humans (14). Baseline R_L_ remained well within the normal range both before and after lidocaine nebulization (Table 5). Throughout HBP, tidal volume increased in 5/6 horses without clinical signs of reaction. This pattern is commonly seen in EA, as an increased work of breathing causes affected horses to breathe more deeply. Conversely, one horse showed a precipitous decline in TV and clinical evidence of reactivity (increased respiratory effort, cough) when R_L_ doubled following nebulization with 4 mg/mL histamine both pre-and post- lidocaine nebulization. One additional horse was classified as hyperresponsive following lidocaine nebulization based on PC_35_C_dyn_, and one based on PC_75_R_L_ (this was not the same horse). Two of the four horses that had airway hyperresponsiveness post-nebulization showed mild increases in respiratory rate exceeding 20 breaths per minute and declines in tidal volume prior to cessation of bronchoprovocation. As C_dyn_ is a frequency-dependent variable, it will decrease as respiratory rate increases and tidal volume decreases, and suggests non-homogeneous ventilatory distribution, both likely contributing to a diagnosis of airway hyperresponsiveness in these patients. The largest percent reduction of PC35_Cdyn_ and PC75_RL_ occurred in the non-hyperresponsive group. In human patients, reductions in forced expiratory volume in 1 second (FEV_1_) were appreciated in as little as 2 min after lidocaine nebulization, and previous response to HBP was not a good predictor of whether patients would have bronchoconstriction after nebulization with lidocaine (49). Moreover, lidocaine inhalation attenuated histamine-induced bronchospasm in other human studies (50). In contrast to the significant changes observed in initially non-hyperresponsive horses in this study by either measure, lidocaine nebulization has been reported to cause initial bronchoconstriction in humans with baseline bronchial hyperreactivity (51), which was subsequently shown to be dose-related with progressive decreases in FEV_1_ (52). However, the lidocaine-induced initial decrease in FEV_1_ reported (-3% to−7.5%) did not meet the threshold for clinical significance in human obstructive airway disease (decrease of > 15%). There is some evidence that local anesthetics, such as lidocaine, may block essential neurogenic bronchodilatory responses (49, 53). Pretreatment with bronchodilators, which is commonly recommended clinically to maximize drug delivery to the distal airways, prevents initial bronchoconstriction in humans (54), and could prove useful in subsequent studies of nebulized lidocaine in horses to attempt to ameliorate the observed bronchoconstriction. Additionally, studies to identify the duration of AHR following single nebulization, and possible persistence or escalation of this phenomenon with repeat administration will prove essential when evaluating the viability of this medication for use in EA.

Preservative-free lidocaine nebulization resulted in neither quantitative nor qualitative changes in BAL cytology performed immediately post-nebulization with a single dose. This finding is supported in other species, where single dosing in guinea pigs and humans failed to alter BAL cytology (55, 56). While no deleterious effects were recorded following single dose nebulization across species, positive results have been appreciated in continued use studies. Twice daily lidocaine treatment for 2 weeks resulted in decreased BAL neutrophilia in a clinical trial of equine asthmatics (6). Previous work has raised the question of the effect of lidocaine on BAL eosinophilia: while 1 week of lidocaine inhalation decreased the total number of cells in BALF, the proportion of BAL eosinophils, and eosinophilic infiltration in the airway walls of ovalbumin challenged guinea pigs (55), chronic lidocaine nebulization did not significantly alter BAL eosinophilia in healthy or ovalbumin exposed cats (5). As only one horse in our current study had an excessive percentage of eosinophils, the question remains unanswered. There is no suspicion of diluting such inflammatory cells with serial sampling, or exacerbating inflammation with serial HBP, as previous investigations have shown no effect on BAL cytology following HBP or sequential BALs within a 24-h period (57–59). While the effect of serial lidocaine exposure on BAL cytology remains to be evaluated, no immediate idiosyncratic effect on lower airway cytology was noted.

Before clinical recommendations can be made, additional data is needed to outline the safety, drug delivery, and immediate response to nebulized lidocaine in clinically affected equine asthmatics. Namely, the effect of repeated nebulized lidocaine treatment on BAL cytology and cumulative drug concentrations in both BAL and plasma remain to be evaluated within affected horses. Also, due to the small sample size in the current study, it is possible that statistical assessment failed to reject the null hypothesis, thus underreporting effects of the nebulized drug. As four of the horses assessed showed abnormalities in either TMS, HBP, or BAL cytology on baseline assessment, the implication of these findings in clinically normal horses not enrolled in an exercise program must be considered. Patient factors such as depth of breathing, airway reactivity, bronchospasm, coughing, and increased membrane permeability make it likely that nebulized lidocaine would reach lower concentrations in the distal airways of equine asthmatics, while a greater proportion of delivered drug would be absorbed systemically. Beyond safety, clinical trials using nebulized lidocaine are needed to determine the dose and duration of treatment necessary to decrease or resolve AHR in affected horses. The low systemic concentrations reached with 1 mg/kg nebulized lidocaine indicate a reasonable margin of safety should higher doses be needed to achieve a therapeutic effect.

## Conclusions

Nebulization of 1 mg/kg 4% preservative-free lidocaine in healthy horses reached acceptable concentrations in the lower airways while having modest systemic absorption and no immediate adverse effect. Serum and urine lidocaine levels remained detectable for 24 and 48 h respectively following nebulization of a single dose. No immediate inflammatory changes were appreciated on BAL cytology, and minimal laryngeal hyposensitization was observed. While baseline lung resistance was unchanged following lidocaine nebulization, AHR was appreciated in a subset of horses, classified as increased response to HBP following nebulization of lidocaine in the absence of clinical signs. While drug deposition and systemic absorption in equine asthmatics remains unknown, and therapeutic dose and duration recommendations remain to be elucidated, lidocaine dosed at 1 mg/kg appears to be a safe and well-tolerated medication for nebulization in the healthy horse.

## Data availability statement

The datasets generated for this study are available on request to the corresponding author.

## Ethics statement

The animal study was reviewed and approved by the Institutional Animal Care and Use Committee (IACUC) and Clinical Sciences Research Committee (CSRC) at Cummings School of Veterinary Medicine. Written informed consent was obtained from the owners of owned animals for their participation in this study.

## Author contributions

JM, DB, and MM were involved in the experimental design, data collection, analysis, and manuscript writing. MC and IZ performed pharmacokinetic assessment and interpretation, while MB designed and validated assays for measurement of lidocaine and MEGX. All authors contributed to the article and approved the submitted version.

## Funding

The Flexineb nebulizer systems used in this study were previously donated by Flexineb North America.

## Conflict of interest

The authors declare that the research was conducted in the absence of any commercial or financial relationships that could be construed as a potential conflict of interest.

## Publisher's note

All claims expressed in this article are solely those of the authors and do not necessarily represent those of their affiliated organizations, or those of the publisher, the editors and the reviewers. Any product that may be evaluated in this article, or claim that may be made by its manufacturer, is not guaranteed or endorsed by the publisher.

## Abbreviations

α: rate constant of distribution phase
β: rate constant of elimination phase compartment
λ: elimination rate constant
A: extrapolation to time 0 of the distribution phase
ACN: acetonitrile
AHR: airway hyperreactivity
ARCI: Association of Racing Commissioners International
AUC_0−∞_: area under the plasma concentration time curve from 0 to infinity
AUMC_0−∞_: area under the first moment curve from 0 to infinity
B: extrapolation to time 0 of the elimination phase
BAL: bronchoalveolar lavage
BALF: bronchoalveolar lavage fluid
C_0_: initial concentration
C_dyn_: dynamic compliance
C_ELF_:C_maxplasma_: ratio of the estimated concentration in the epithelial lining fluid compared to the maximum concentration in the plasm
C_max_: maximum concentration
CL: systemic clearance
CL/F: apparent clearance
CSRC: Clinical Studies Review Committee
dPpl: change in pleural pressure
EA: equine asthma
ELF: epithelial lining fluid
Eos: eosinophil
F: bioavailability
FEV_1_: forced expiratory volume in 1 second
GM-CSF: granulocyte macrophage colony stimulating factor
GS1: nebulizer gas
GS2: auxiliary gas
HBP: histamine bronchoprovocation
HPLC: high-performance liquid chromatography
IACUC: Institutional Animal Care and Use Committee
IFN: interferon
IL: interleukin
IS: ion spray voltage
K_10_: microconstant representing drug absorption into the central compartment
K_12_ and K_21_: microconstants representing distribution between the central and peripheral (2^nd^)
LOD: limit of detection
LLOQ: lower limit of quantification
Mast: mast cell
MAT: mean absorption time
MEGX: monoethylglycinexylidide
MRT: mean residence time
PC_35_C_dyn_: provocative concentration of histamine where dynamic compliance decreases by 35
PC_50_R_L_: provocative concentration of histamine where lung resistance increases by 50%
PC_75_R_L_: provocative concentration of histamine where lung resistance increases by 75%
PC_100_R_L_: provocative concentration of histamine where lung resistance increases by 100%
PMN: neutrophil
R_L_: lung resistance
SAA: serum amyloid A
T_1/2_: half-life
t_½α_: distribution half-life
t_½β_: elimination half-life
t_½λ_: elimination half-life
T_max_: time to reach the maximum observed drug concentration
TMS: tracheal mucus score
TV: Tidal volume
V_c_: volume of distribution of the central compartment
V_dβ_: volume of distribution during the elimination phase
V_dss_: steady-state volume of distribution
V_z_/F: apparent volume of distribution.

## Appendix

**Table A:** Pulmonary concentrations of lidocaine after nebulization of lidocaine 1mg/kg in 6 healthy horses.

**Parameter**	**Mean ± SD**
BAL volume saline infused (mL)	516.70 ± 40.82
BAL yield volume (mL)	289.17 ± 65.83
Serum Urea (mg/dL)	34.24 ± 6.83
BALF Urea (mg/dL)	0.56 ± 0.14
BALF Lidocaine Concentration (ng/mL)	142.83 ± 34.25
ELF Dilution Factor (%)	1.71 ± 0.54
ELF volume (mL)	4.94 ± 1.78
Lidocaine C_ELF_ (μg /mL)	9.63 ± 5.05

BAL, bronchoalveolar lavage; BALF, bronchoalveolar lavage fluid; ELF, epithelial lining fluid; C_ELF_, concentration in the epithelial lining fluid.
